# The clinical significance of endoplasmic reticulum stress related genes in non-small cell lung cancer and analysis of single nucleotide polymorphism for CAV1

**DOI:** 10.3389/fmolb.2024.1414164

**Published:** 2024-08-06

**Authors:** Shuang Li, Junting Chen, Baosen Zhou

**Affiliations:** Department of Clinical Epidemiology and Center of Evidence-Based Medicine, The First Hospital of China Medical University, Shenyang, China

**Keywords:** non-small cell lung cancer, endoplasmic reticulum stress, single nucleotide polymorphisms, CAV1, bioinformatics

## Abstract

In recent years, protein homeostasis imbalance caused by endoplasmic reticulum stress has become a major hallmark of cancer. Studies have shown that endoplasmic reticulum stress is closely related to the occurrence, development, and drug resistance of non-small cell lung cancer, however, the role of various endoplasmic reticulum stress-related genes in non-small cell lung cancer is still unclear. In this study, we established an endoplasmic reticulum stress scores based on the Cancer Genome Atlas for non-small cell lung cancer to reflect patient features and predict prognosis. Survival analysis showed significant differences in overall survival among non-small cell lung cancer patients with different endoplasmic reticulum stress scores. In addition, endoplasmic reticulum stress scores was significantly correlated with the clinical features of non-small cell lung cancer patients, and can be served as an independent prognostic indicator. A nomogram based on endoplasmic reticulum stress scores indicated a certain clinical net benefit, while ssGSEA analysis demonstrated that there was a certain immunosuppressive microenvironment in high endoplasmic reticulum stress scores. Gene Set Enrichment Analysis showed that scores was associated with cancer pathways and metabolism. Finally, weighted gene co-expression network analysis displayed that CAV1 was closely related to the occurrence of non-small cell lung cancer. Therefore, in order to further analyze the role of this gene, Chinese non-smoking females were selected as the research subjects to investigate the relationship between CAV1 rs3779514 and susceptibility and prognosis of non-small cell lung cancer. The results showed that the mutation of rs3779514 significantly reduced the risk of non-small cell lung cancer in Chinese non-smoking females, but no prognostic effect was found. In summary, we proposed an endoplasmic reticulum stress scores, which was an independent prognostic factor and indicated immune characteristics in the microenvironment of non-small cell lung cancer. We also validated the relationship between single nucleotide polymorphism locus of core genes and susceptibility to non-small cell lung cancer.

## 1 Introduction

Lung cancer is the main cause of cancer-related deaths, accounting for approximately 18% of all cancer deaths worldwide (Sung et al., 2021). It is estimated that nearly 1.8 million people die from lung cancer, which means that one in every five cancer patients dies from lung cancer (Sung et al., 2021). Lung cancer mainly includes two histological subtypes, small cell lung cancer (SCLC) and non-small cell lung cancer (NSCLC), with NSCLC accounting for about 85% of the total number of lung cancer cases (Herbst et al., 2018). Lung adenocarcinoma (LUAD) and lung squamous cell carcinoma (LUSC) are the most common subtypes in the NSCLC (Thai et al., 2021). The main treatment for early non metastatic NSCLC in clinical practice is radical surgical resection (Donington et al., 2011). However, due to the insidious onset of NSCLC and the lack of specific clinical manifestations, most patients are already in local advanced or distant metastasis at the time of diagnosis (Blandin Knight et al., 2017), synchronous combination radiotherapy and chemotherapy has always been the preferred method for treating unresectable advanced NSCLC in recent years, demonstrating moderate survival benefits (Duma et al., 2019). In addition, multiple studies have shown that emerging immunotherapies can also significantly improve the overall survival of advanced NSCLC patients (Borghaei et al., 2021; Brahmer et al., 2023). However, even with the continuous development of modern medical technology and significant improvements in diagnostic and treatment techniques, the 5-year survival rate of NSCLC is still relatively low. According to statistics, the overall 5-year survival rate of NSCLC patients still does not exceed 15% (Molinier et al., 2020). The 5-year survival rate of NSCLC is closely related to the clinically pathological stage it is in. As the stage progresses, the 5-year relative survival rate of NSCLC ranges from 82% in stage I patients to 59% in stage II patients, and gradually decreases to 16% in stage III patients and 10% in stage IV patients (Jeon et al., 2023), Therefore, early diagnosis of NSCLC is crucial for improving patient prognosis.

The endoplasmic reticulum is an efficient and complex membrane-bound organelle in eukaryotes, organized into multiple functional zones that can regulate various biological functions, including controlling the folding and modification of protein spatial structure, biosynthesis and metabolism of lipids, storage and release of calcium ions, and intercellular communication (Lemmer et al., 2021). More than one-third of the proteins required by cells are assembled and secreted through the endoplasmic reticulum, and quality control is carried out to promote effective protein folding and transportation to maintain protein homeostasis (Adams et al., 2019). Under the influence of various factors such as hypoxia, acidosis, nutrient deficiency, and drastic changes in temperature, changes in intracellular homeostasis occur, leading to damage to the folding ability of endoplasmic reticulum proteins (Oakes, 2020). Unfolded or misfolded proteins accumulate in the lumen, causing the separation of the molecular chaperone binding protein BiP (also known as GRP78) from the three sensors, triggering an endoplasmic reticulum stress state marked by unfolded protein response (UPR), which reduces protein synthesis and increases degradation of related proteins in order to reduces cellular load (Almanza et al., 2019; Hetz et al., 2020). But when the stress level is severe, the duration is too long, and the adaptive UPR cannot restore intracellular homeostasis, cell death can occur under the joint action of multiple pathways (Kapuy et al., 2020).

Research has shown that although endoplasmic reticulum stress plays an important role in the occurrence and development of lung cancer, it also demonstrates its potential as a therapeutic target for lung cancer. The incidence and expression level of endoplasmic reticulum stress protein nuclear translocation significantly increase with the progression of atypical adenomatous hyperplasia from low to high levels and then to LUAD. This may indicate that endoplasmic reticulum stress is involved in the development of lung cancer from benign stages to malignant or invasive stages (Ogata et al., 2019). The overexpression of X-box Binding Proteins (XBP1s) generated in response to endoplasmic reticulum stress can not only promote the invasion and metastasis of NSCLC cells *in vivo* and *in vitro* by upregulating IGFBP3 (Luo et al., 2021), but also possible to induce metabolic remodeling by inducing the expression of PDK1, thereby inducing the accumulation of lactate, activating the epithelial mesenchymal transition process of NSCLC, and promoting the progression of cancer cells (Mao et al., 2022). Other studies have shown that the absence of an important endoplasmic reticulum chaperone and master regulator of endoplasmic reticulum functions, BiP, in alveolar epithelial cells can inhibit Kras gene-driven lung cancer, significantly prolong survival, and reduce the activation of cancer cell viability and apoptotic markers (Rangel et al., 2021). *In vitro* cell experiments have shown that ginsenoside extract upregulates autophagic flux in NSCLC by activating the downstream rapamycin target protein signaling pathway of endoplasmic reticulum stress, ultimately leading to time-dependent and concentration dependent cell autophagic death (Zhao et al., 2019). Myricetin activates Caspase-3 through the endoplasmic reticulum stress pathway, triggering cell pyroptosis in NSCLC. This not only significantly reduces cell viability *in vitro* experiments, but also significantly slows down the growth rate of lung cancer *in vivo* experiments (Han et al., 2022). Furthermore, curcumin (Wang et al., 2022), icariside II (Tang et al., 2022) and regorafenib (Sui et al., 2023) both can increase the sensitivity of NSCLC to cisplatin and increase the apoptosis rate of cancer cells by promoting endoplasmic reticulum stress signal transduction, elucidating that endoplasmic reticulum stress may become a target for overcoming cisplatin resistance in NSCLC in the future.

Epidemiological investigations have confirmed that smoking is the most important risk factor for lung cancer, and the risk of lung cancer is positively correlated with the number and duration of smoking, and negatively correlated with the age at which smoking begins (Hansen et al., 2018). Cohort studies have shown that an increase of 10 packs of smoking per year will lead to a 43% and 64% increase in the risk of lung cancer for males and females, respectively (Hansen et al., 2018). However, the proportion of non-smokers with NSCLC is steadily increasing year by year, accounting for 11.45% of all NSCLC patients as a whole, with over 65% of non-smokers having NSCLC composed of females (Pelosof et al., 2017), A clinical data published in the Britain in the same year also found a similar trend (Cufari et al., 2017). This proportion of the females in China is even higher compared to other countries (Pinheiro et al., 2022), indicating that genetic factors may play a significant role in the etiology of lung cancer in non-smoking females in China.

As one of the most common heritable forms of variation in humans, single nucleotide polymorphism mainly refers to the polymorphism of DNA sequences caused by a single nucleotide change frequency exceeding 1% in the human genome (Brookes, 1999). In recent years, researchers have extensively explored the relationship between single nucleotide polymorphism and susceptibility and prognosis of NSCLC (Dimitrakopoulos et al., 2021; Andrzejczak et al., 2022; Du et al., 2022), however, these studies are still far from sufficient for NSCLC with a heavy disease burden, and more research is needed to further explore the reasons for its susceptibility and prognostic changes.

Therefore, in this study, we established an endoplasmic reticulum stress score, which not only accurately predicts the prognosis of NSCLC patients, but also distinguishes different clinical features, immune infiltration, tumor mutation burden, signaling pathways, and drug sensitivity of NSCLC. In addition, this study established a nomogram based on endoplasmic reticulum stress scores, combining with clinical features, and found that it performed well in estimating 1-year, 3-year, and 5-year survival rates of NSCLC patients, while bringing significant clinical net benefits to patients at 3 years, Meanwhile, it was also found that CAV1, which participated in the construction of endoplasmic reticulum stress scores, may be the core gene involved in the occurrence and development of NSCLC. Therefore, single nucleotide polymorphism analysis of this gene was conducted to explore its role in the occurrence and development of NSCLC.

## 2 Materials and methods

### 2.1 Data collection

We downloaded gene expression and clinical data of TCGA-LUAD and TCGA-LUSC (http://cancergemome.nih.gov/), deleted non expressed genes and samples with missing survival time and survival status. After adjusting batch effects using “ComBat” in the “SVA” package (Leek et al., 2012), we merged them into the NSCLC gene expression dataset. A total of 943 cancer samples and 107 normal adjacent tissue samples were included in the study. The data from GSE41271 and GSE42127 in the GEO database (http://www.ncbi.nlm.nih.gov/geo/), totaling 438 NSCLC cancer samples, were also subjected to the same correction process to form a validation dataset. We Searched the Genecards (https://www.genecards.org/) using the keyword “Endoplasmic Reticulum Stress” to download endoplasmic reticulum stress-related genes. To ensure good correlation, genes with scores ≥7 were extracted, and a total of 785 mRNA were included in the analysis.

### 2.2 GO and KEGG analysis

The Gene Ontology (GO) biological processes and Kyoto Encyclopedia of Genes and Genomes (KEGG) pathways were used to perform functional enrichment analysis on the differentially expressed endoplasmic reticulum stress genes, in order to obtain the potential functions and signaling pathways of the genes, and to verify whether the analyzed genes are closely related to endoplasmic reticulum stress. Both enrichment analysis were completed using the clusterProfiler package (Yu et al., 2012), with screening conditions of adjust-p and adjust-q both < 0.05.

### 2.3 Construction and validation of ERSS

We performed univariate Cox regression analysis on genes to identify genes that were significantly correlated with prognosis. Next, Endoplasmic Reticulum Stress Scores (ERSS) was defined by using principal component analysis, this method had advantage of focusing the score on the set with the largest block of well correlated (or anticorrelated) genes in the set, while down-weighting contributions from genes that do not track with other set members. We then defined the ERSS using a method similar to GGI (Sotiriou et al., 2006; Zeng et al., 2019; Bai et al., 2021).

ERSS=∑PC1A−∑PC1B

Among them, A represented the gene cluster with a positive Cox coefficient in the univariate Cox analysis results, and B represented the gene cluster with a negative Cox coefficient. ΣPC1A represents the PC1 score of the A gene cluster, as does ΣPC1B. Using the same formula used for the train set, we calculated ERSS for patients in the validation cohort. ROC curves were used to test the predictive performance of ERSS in predicting survival in different years.

### 2.4 Survival analysis

The study used the “surv_cuttpoint” function in the “survminer” package to calculate the optimal cutoff value of ERSS. The ERSS was divided into high and low groups, and Kaplan-Meier survival analysis was used for survival analysis and logrank test was used for significance test. Then apply univariate and multivariate Cox regression analysis to analyze whether ERSS was an independent prognostic factor.

### 2.5 Immune microenvironment analysis

The various types of immune cells and stromal cells infiltrating the tumor microenvironment constituted a part of the cells in tumor tissue, which not only disrupted tumor signals at the internal molecular level, but also played a crucial role in biological behaviors such as cancer progression and invasion (de Visser and Joyce, 2023), Therefore, analyzing the tumor microenvironment simultaneously was more helpful in understanding the pathogenesis of tumors. ssGSEA was used for quantitative analysis of the relative abundance of immune infiltrating cells in NSCLC. In addition, the ESTIMATE estimated the overall immune scores, stroma scores, and tumor purity based on gene expression in tumor tissue.

### 2.6 Tumor mutation burden analysis

TMB was defined as the total number of somatic mutations per million nucleotides, which is an indicator of the frequency of gene mutations. It could significantly increase the sensitivity of immunotherapy in PD-L1 overexpressing subgroups and had become an important marker for predicting the efficacy of immunotherapy in NSCLC in recent years (Negrao et al., 2021; Ricciuti et al., 2022).

### 2.7 Construction and validation of nomogram

We constructed a nomogram using the R package “rms” and “survival”, combined with ERSS and currently known factors that affect the likelihood of patient survival in clinical practice (including age and pathological stage), to predict the 1-year, 3-year, and 5-year survival probabilities of NSCLC patients, and evaluated the reliability of the nomogram using C-index and calibration chart. Simultaneously, we established a decision curve analysis to compare the net benefits obtained from two strategies: nomogram and commonly used clinical decision-making.

### 2.8 Gene Set Enrichment Analysis

This study aimed to explore whether different biological pathways were involved in the occurrence and development of NSCLC in the high and low ERSS groups, using GSEA_ 4.1.0 to conduct Gene Set Enrichment Analysis (GSEA) on gene expression data of NSCLC. We used the high and low levels of ERSS as the phenotype, and the gene sets datasets were “c2. cp.kegg.v2023.1. Hs.symbols.gmt”and“c5. go.v2023.1. Hs.symbols.gmt”, numbers of permutations were 1000. It is generally believed that pathways with |NES| > 1, NOM *p*-value < 0.05, and FDR q-value < 0.25 were significantly enriched.

### 2.9 Drug sensitivity prediction

In order to evaluate whether endoplasmic reticulum stress affects patient sensitivity to drugs in clinical treatment of NSCLC, this study used pRRorphic packages to predict the median inhibitory concentration (IC50) of drugs, and thus estimated drug sensitivity for each patient (Geeleher et al., 2014), including conventional drugs such as gefitinib, erlotinib, cisplatin, and paclitaxel recommended in the AJCC guidelines for the treatment of NSCLC.

### 2.10 Weighted gene co-Expression network analysis

Weighted Gene Co-Expression Network Analysis (WGCNA) was a scale-free gene network analysis method that utilized soft thresholds β to amplified the differences in gene correlation strength in order to obtain gene modules with co expression, which was more suitable for analyzing the regulation of genes in organisms. At the same time, the visualization of the correlation between co expression modules and external traits of samples can be completed through WGCNA (Langfelder and Horvath, 2008). Therefore, we fully utilized WGCNA to search for gene sets closely related to the target trait to be analyzed, and discusses whether this gene set contains core genes related to endoplasmic reticulum stress.

### 2.11 Collection of research subjects and epidemiological investigation

Approved by the Ethics Committee from the First Hospital of China Medical University No. [2022] 477, all cases and control venous blood were included in the blood sample library of the Evidence-Based Medicine Center. The inclusion criteria for cases included 1) a new pathological diagnosis of NSCLC according to diagnostic criteria; 2) Non-smoking females:female patients who had smoked fewer than 100 cigarettes at the time of diagnosis of non-small cell lung cancer; 3) Radiotherapy had not been adopted for treatment at the moment. Exclusion criteria included 1) non primary NSCLC; 2) Failed to obtain smoking history information. The control group was healthy non-smoking females. A total of 467 NSCLC cases and 395 controls were included in the final sample library, with a total of 862 study subjects included in this case-control study. 1 mL of the whole blood was extracted from each study subject from the sample library for experimentation.

Non-smokers were defined as individuals who had smoked less than 100 cigarettes at the time of diagnosis of NSCLC, passive smokers were defined as individuals who had been exposed to smoke from one or more cigarettes per day for at least 1 year, and those who had been exposed to cooking oil were defined as individuals who experienced discomfort symptoms more than twice a week when their eyes or throat were stimulated by the oil while cooking in the kitchen.

### 2.12 DNA purification and quality control

The commonly used phenol chloroform method was used to separate DNA from the whole blood, and the Taqman probe method was used for genotyping. The reaction system includes:1 μL DNA, 2.5 μL Taqman Master Mix, 1.2 μL ddH_2_0, 0.1 μL ROX and 0.2 μL probe primers. The PCR was conducted under the following conditions: Pre denaturation at 95°C for 10 min, denaturation at 92°C for 30 s, annealing and elongation at 60°C for 1 min, and a total of 47 cycles. Due to differences in fluorescence signal intensity between FAM and VIC at different alleles, the analysis results of genotype identification were identified by SDS software.

### 2.13 Statistic analysis

The visualization and statistical analysis of graphics were completed by R4.0.3 and R4.2.2. The Chi-square test and Fisher’s exact test were used to analyze the high and low distribution of ERSS in different clinical features. The Wilcoxon test was used to compare the differences between two groups of features, and the Kruskal Wallis test was used to compare whether there were differences in the score distribution of multiple groups of samples. The correlation between the two continuous variables was calculated through linear correlation regression. The Odd Ratios (OR) and its 95% confidence interval (CI) were calculated using unconditional logistic regression to evaluate the relationship between rs3779514 and susceptibility to NSCLC, as well as the multiplicative interaction between cooking oil exposure and rs3779514. The calculation of the additive interaction between cooking oil fume exposure and rs3779514 was carried out using an automated calculation table developed by Andersson et al. (2005), When 0 was not within the 95% CI of Attributable Proposition Due To Interaction (AP) or Relative Excess Risk Due To Interaction (RERI), or when 1 was not included in the Synergy Index S) 95% CI, it indicated that there was an additive interaction between the gene and the environment. All statistical results indicate significant statistical significance when *p* < 0.05 is met.

## 3 Results

### 3.1 Differentially expressed endoplasmic reticulum stress genes

Firstly, a preliminary analysis was conducted on the characteristics of NSCLC data in the screened TCGA. The results showed that the proportion of male and female patients in lung adenocarcinoma was similar (45.78% for males and 54.22% for females). In lung squamous cell carcinoma, the number of male patients was approximately three times that of female patients (73.99% for males and 26.01% for females), which roughly conforms to the gender distribution trend in NSCLC (Meza et al., 2015). Next, differential analysis was performed on 943 NSCLC samples and 107 normal samples. The results showed a total of 2330 differentially expressed genes (*p* < 0.05), of which 946 genes were upregulated and 1384 genes were downregulated in the NSCLC samples. By intersecting the differentially expressed genes with the collected endoplasmic reticulum stress-related genes, a total of 97 differentially expressed endoplasmic reticulum stress-related genes were obtained.

### 3.2 GO and KEGG analysis

**FIGURE 1:**
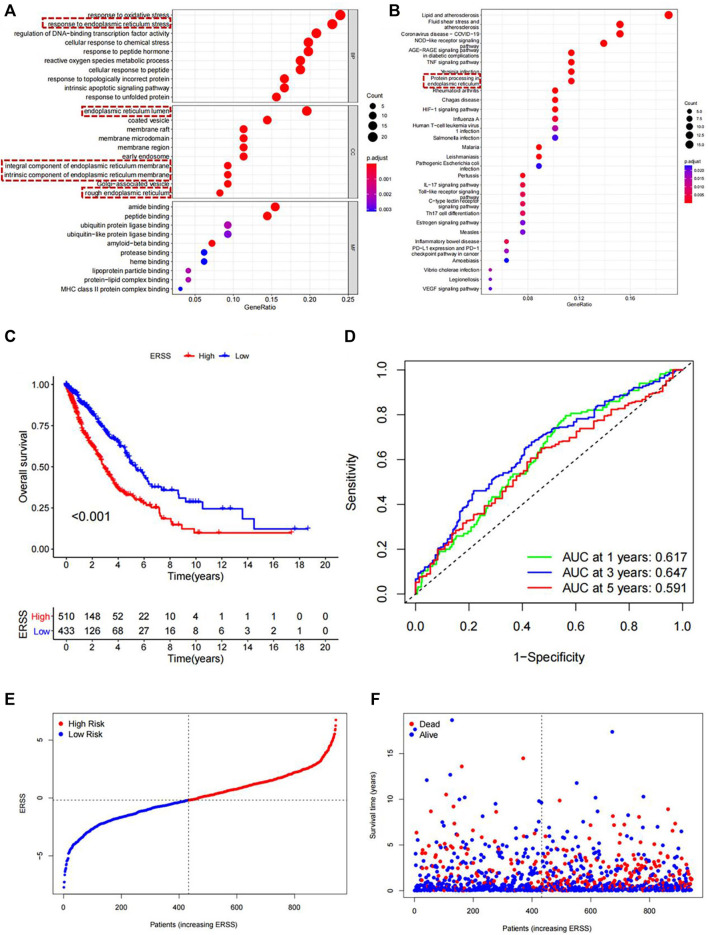
Functional enrichment analysis and Establishment of ERSS for NSCLC. **(A)** GO analysis of 97 differentially expressed endoplasmic reticulum stress-related genes. **(B)** KEGG analysis of 97 differentially expressed endoplasmic reticulum stress-related genes **(C)** KM curve comparing overall survival between high and low ERSS groups. **(D)** ROC curve of the prognostic characteristics of ERSS. **(E)** Risk plot of NSCLC patients. **(F)** Survival status plot of NSCLC patients.

In order to conduct a more in-depth analysis of the potential core functions and pathways of 97 differentially expressed endoplasmic reticulum stress-related mRNA at the biological functional level, GO and KEGG analysis were performed on them. As shown in Figures 1A, B, the pathways for ranking in the top ten of three different parts in GO and the top thirty in KEGG were shown. The most noteworthy pathway of this study was that in the GO results (Figure 1A), the “response to endoplasmic reticulum stress” pathway was significantly enriched in biological processes, At the same time, pathways enriched in the cellular components included the “endoplasmic reticulum lumen”, “integral component of the endoplasmic reticulum membrane”, “intrinsic components of the endoplasmic reticulum membrane”, and “rough endoplasmic reticulum”. A total of 5 pathways related to endoplasmic reticulum stress were enriched in GO analysis. In addition, the KEGG results also indicated (Figure 1B) that the “protein processing in endoplasmic reticulum” was significantly enriched. The above results demonstrated that the screened genes were closely related to endoplasmic reticulum stress.

### 3.3 Screening of prognostic genes and establishment of ERSS

Through univariate Cox regression analysis, 14 genes significantly correlated with the prognosis of NSCLC were obtained from the intersection genes, of which 12 genes were prognostic risk factors for NSCLC and 2 genes were prognostic protective factors for NSCLC (*p* < 0.05). Considering the complexity and heterogeneity of endoplasmic reticulum stress-related genes in different individuals, a PCA algorithm was used to define ERSS based on these 14 prognostic related genes to quantify endoplasmic reticulum stress in each patient, we then defined the ERSS using a method similar to GGI (Sotiriou et al., 2006; Bai et al., 2021).

### 3.4 Kaplan-Meier survival analysis

This study used the optimal cutoff value to divide ERSS into high and low groups, and compared the overall survival of NSCLC patients in the high and low groups. The KM survival curve intuitively showed that the overall survival rate of the high ERSS group was significantly lower than that of the low ERSS group (*p* < 0.001, Figure 1C), indicating that high ERSS was a risk factor for NSCLC survival. Next, the time-dependent ROC curve was used to evaluate the predictive reliability of prognostic features (Figure 1D), where the areas under the curves for 1 year, 3 years, and 5 years were 0.617, 0.647, and 0.591, respectively. At the same time, the risk plot and survival status plot indicated that as ERSS increased, the number of deaths also increased significantly (Figures 1E, F), indicating that ERSS was a good indicator for predicting the overall survival rate of NSCLC patients.

### 3.5 Correlation between ERSS and clinical features

In order to explore the correlation between ERSS and different clinical features, this study used the Chi-square test and Fisher’s exact test to preliminarily analyze whether the proportion of high and low ERSS in different clinical features was different (Supplementary Table S1). The results showed that the distribution of high and low ERSS was significantly different in gender, pathological stage, T stage, N stage, smoking, and status (*p* < 0.05).

**FIGURE 2:**
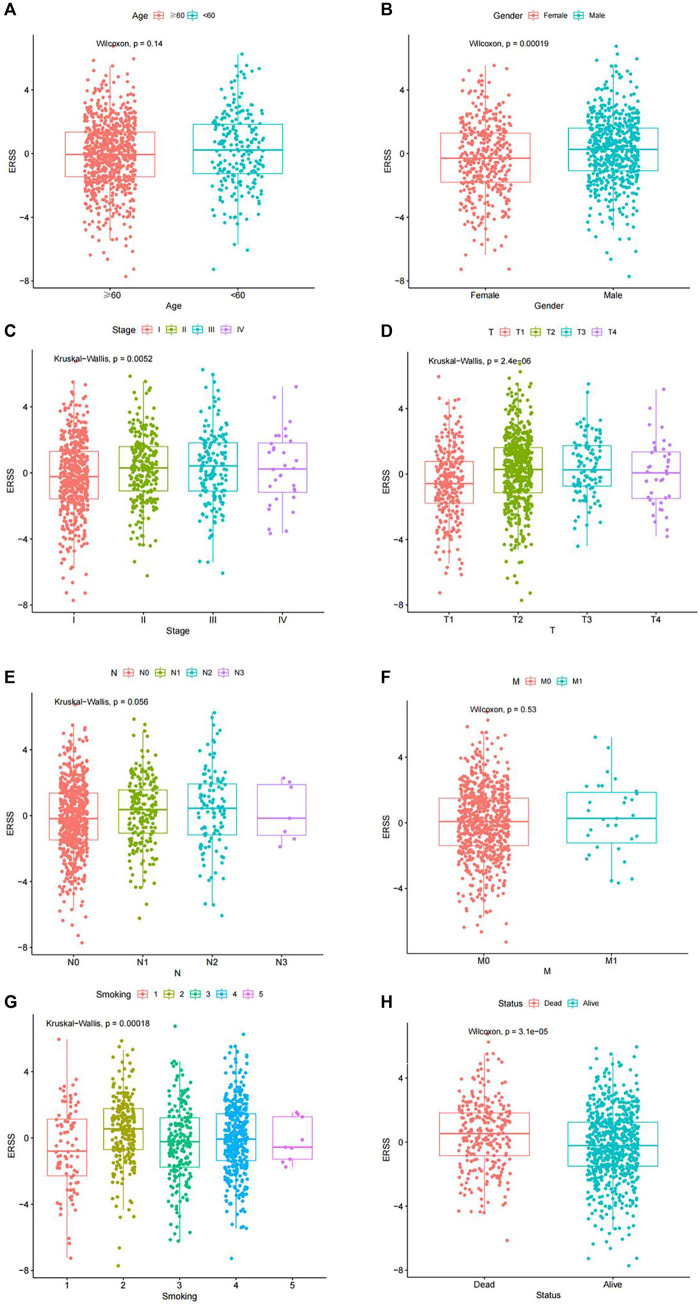
Distribution of ERSS in NSCLC patients of clinical features. **(A)** Distribution of ERSS in NSCLC patients of different ages. **(B)** The distribution of ERSS in NSCLC patients of different genders. **(C)** The distribution of ERSS in NSCLC patients with different pathological stages. **(D)** The distribution of ERSS in NSCLC patients in different T stages. **(E)** The distribution of ERSS in NSCLC patients with different N stages. **(F)** The distribution of ERSS in NSCLC patients with different M stages. **(G)** The distribution of ERSS in NSCLC patients with different smoking histories, where the number “1–5″ represented the same meaning as Supplementary Table S1. **(H)** The distribution of ERSS in NSCLC patients with different survival status.

In order to clarify the distribution and differences of ERSS in different clinical features, the Wilcoxon test and the Kruskal Wallis test were performed using a continuous variable of ERSS in different levels of clinical features after removing unknown states. The results were similar to the Chi-square test, indicating that higher ERSS can be obtained in males, advanced pathological stage, advanced T stage, and death status (Figures 2B–D, H, *p* < 0.05), At the same time, the distribution of ERSS in different smoking histories also conformed to the characteristic that ERSS was a survival risk factor in NSCLC. Patients with a smoking history, regardless of whether they quitted smoking or not, showed an increase in ERSS compared to non-smokers, and the overall difference was statistically significant (Figure 2G, *p* < 0.001). However, in the preliminary analysis, N stage with statistical significance lost its significance (Figure 2E).

### 3.6 Cox regression analysis of ERSS and clinical features

**FIGURE 3:**
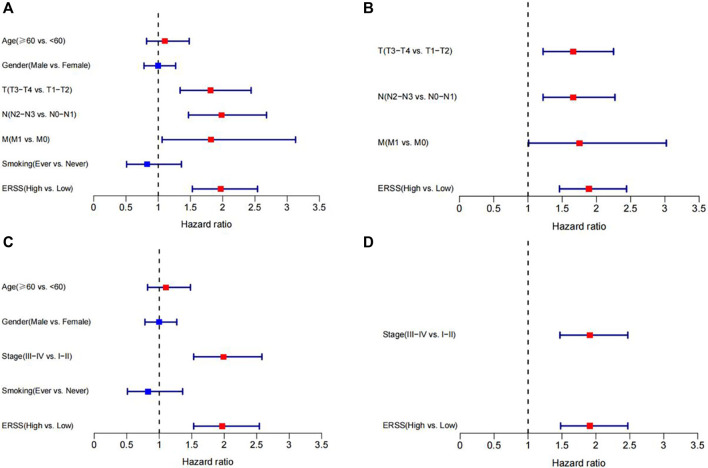
Cox regression analysis of ERSS and clinical features. **(A)** Forest plot of univariate Cox regression analysis results on factors affecting survival in NSCLC patients (including TNM stages). **(B)** Forest plot of multivariate Cox regression analysis results on factors affecting survival in NSCLC patients (including TNM stages). **(C)** Forest plot of univariate Cox regression analysis results on factors affecting survival in NSCLC patients (including pathological stage). **(D)** Forest plot of multivariate Cox regression analysis results on factors affecting survival in NSCLC patients (including pathological stage).

Next, Cox regression analysis will be used to identify factors that affect the prognosis of NSCLC patients. Due to the collinearity between TNM stages and pathological stage, TNM and pathological stage will be divided into two parts and included in Cox regression analysis. The results showed that in univariate Cox regression analysis, the overall survival of NSCLC was significantly correlated with T stage, N stage, M stage, pathological stage, and ERSS; In both models, meaningful variables from univariate Cox regression analysis were included in multivariate Cox regression analysis. After adjusting for other factors, ERSS remained an independent prognostic factor and significantly increased the risk of death in NSCLC patients. The forest plots created based on the analysis results can intuitively observe the effects of various factors (Figure 3).

### 3.7 Validation of ERSS in external dataset

In order to explore whether the established ERSS had the same prognostic effects in other datasets, this study used GEO-NSCLC data from GSE41271 and GSE42127 that belong to NSCLC after correcting for batch effects as the validation dataset. This not only avoided the randomness brought by a single validation dataset, but also ensured sufficient sample size. Using the same ERSS construction method, calculated the ERSS of each NSCLC patient in the validation set. We divided the NSCLC in the validation set into two groups using the optimal cutoff value of ERSS: high and low, used Kaplan Meier survival analysis to verify the impact of ERSS on overall survival. The time-dependent ROC curve was used to evaluate the predictive reliability of ERSS in the validation set, this study applied risk plot and survival status plot to indicate the magnitude of mortality risk for each NSCLC patient. Next, applied the Chi-square test and Fisher’s exact test, and the Wilcoxon test and the Kruskal Wallis test to analyze the correlation between clinical features and ERSS in the validation set. Finally, Cox regression was applied to verify whether ERSS is an independent prognostic factor affecting the survival of NSCLC patients.

#### 3.7.1 Comparison of overall survival between high and low ERSS groups in the validation dataset

**FIGURE 4:**
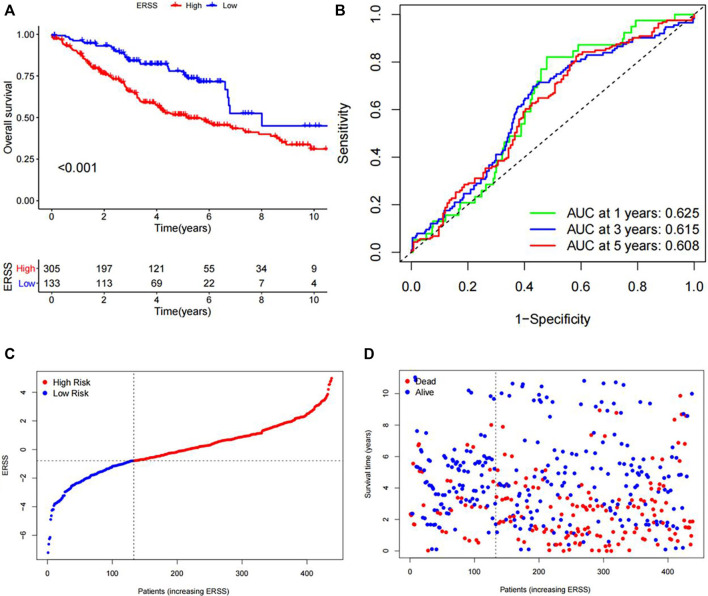
Validation of ERSS for NSCLC. **(A)** KM curve comparing the overall survival between high and low ERSS groups in the GEO validation dataset. **(B)** ROC curve of the prognostic characteristics of ERSS in GEO validation dataset. **(C)** Risk plot of NSCLC patients in GEO validation dataset. **(D)** Survival status plot of NSCLC patients in GEO validation dataset.

The KM analysis results, as shown in Figure 4A, showed that the overall survival of the high ERSS group was significantly lower than that of the low ERSS group (*p* < 0.001). The AUC of the ROC curves at 1 year, 3 years, and 5 years were 0.625, 0.615, and 0.608 respectively, indicating that ERSS had a certain predictive reliability (Figure 4B). At the same time, the risk plot and survival status plot of NSCLC patients in the validation dataset also showed an increase in patient mortality with the increase of ERSS (Figures 4C, D). The above results all confirmed that high ERSS can predict a lower overall survival for patients.

#### 3.7.2 Correlation analysis between ERSS and clinical features in the validation dataset

**FIGURE 5:**
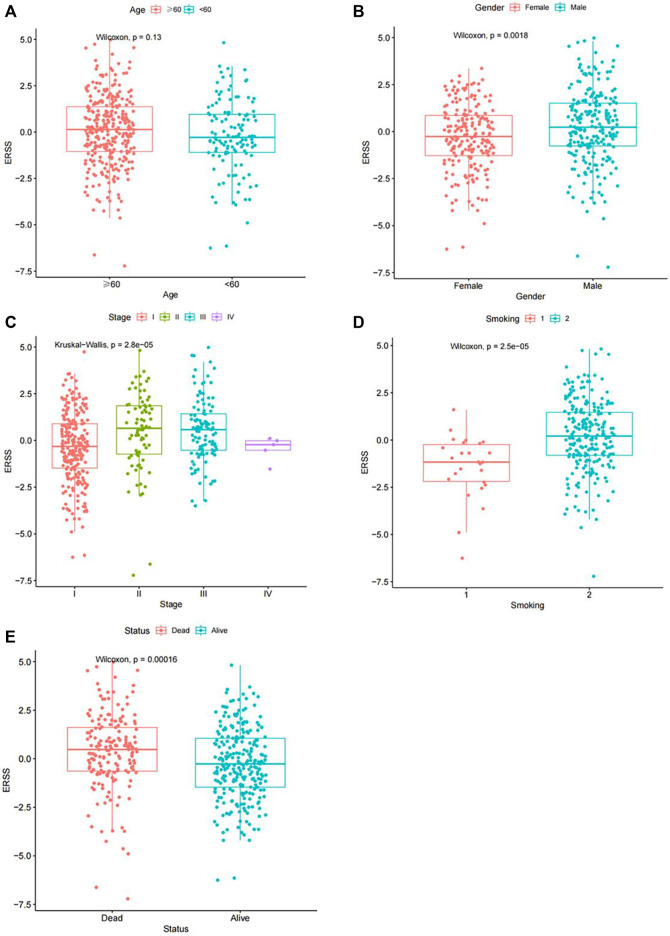
Distribution of ERSS in NSCLC patients of clinical features in the GEO validation dataset. **(A)** Distribution of ERSS in NSCLC patients of different ages in the GEO validation dataset. **(B)** The distribution of ERSS in NSCLC patients of different genders in the GEO validation dataset. **(C)** The distribution of ERSS in NSCLC patients with different pathological stages in the GEO validation dataset. **(D)** The distribution of ERSS in NSCLC patients with different smoking histories in the GEO validation dataset, where the numbers “1, 2″represented the same meaning as Supplementary Table S2. **(E)** The distribution of ERSS in NSCLC patients with different survival states in the GEO validation dataset.

In addition, the Chi-square test and Fisher’s exact test, and the Wilcoxon test and the Kruskal Wallis test all showed that ERSS was closely related to gender, pathological stage, smoking, and status (*p* < 0.05), and higher ERSS was obtained in males, patients with advanced pathological stage, smoking, and death status (Supplementary Table S2, Figure 5).

#### 3.7.3 Cox regression analysis on the correlation between ERSS and clinical features in the validation dataset

**FIGURE 6:**
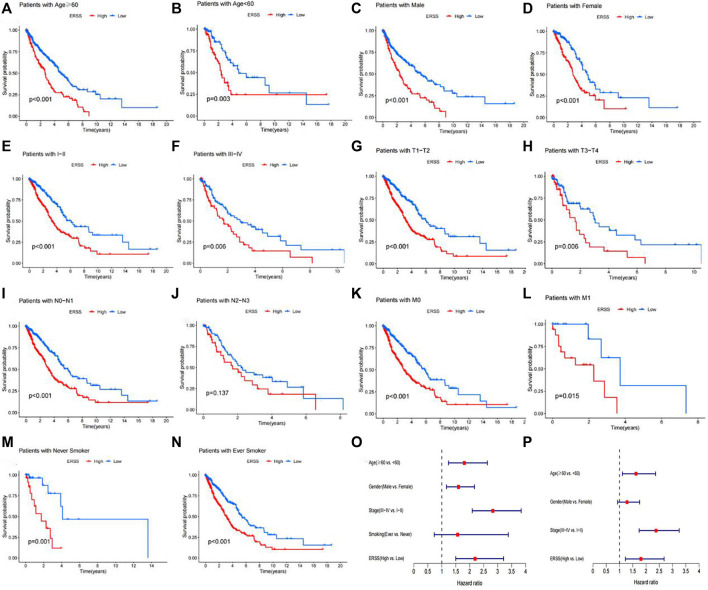
Subgroup analysis of overall survival in NSCLC patients. **(A)** Age ≥60 years. **(B)** Age <60 years. **(C)** Male. **(D)** Female. **(E)** Pathological stage with I-II. **(F)** Pathological stage with III-IV. **(G)** T1-T2 stage. **(H)** T3-T4 stage. **(I)** N0-N1 stage. **(J)** N2-N3 stage. **(K)** M0 stage. **(L)** M1 stage. **(M)** Never smokers. **(N)** Ever smokers. **(O)** Forest plot of univariate Cox regression analysis results on factors affecting survival in NSCLC patients in the GEO validation dataset. **(P)** Forest plot of multivariate Cox regression analysis results on factors affecting survival in NSCLC patients in the GEO validation dataset.

The Cox regression analysis results in the GEO validation dataset showed that age ≥60 years old, male, advanced pathological stage, and ERSS were risk prognostic factors for NSCLC patients in univariate Cox regression analysis (HR > 1, *p* < 0.05). At the same time, the multivariate Cox regression analysis results confirmed that ERSS was still an independent survival risk factor (HR = 1.81, 95% CI = 1.22-2.68, *p* < 0.05). In addition, the Cox regression analysis results of the GEO validation dataset were visually displayed in the forest plot of Figures 6O, P. From then on, the analysis of the GEO validation dataset was completed. In the GEO validation dataset of this study, in addition to verifying that ERSS reduces overall patient survival, the correlation between ERSS and clinical features in the GEO validation dataset was the same as in the TCGA dataset. The prognostic effectiveness of ERSS in different datasets was clearly demonstrated.

### 3.8 Clinical subgroup analysis of ERSS

This study further conducted subgroup survival analysis to determine whether the prognostic model can predict the overall survival of patients based on different clinical features. These patients were separated by age (≥60 years old and <60 years old), gender (male and female), pathological stage (I-II and III-IV), T stage (T1-T2 and T3-T4), N stage (N0-N1 and N2-N3), M stage (M0 and M1), and smoking history (Never and ever) (Figure 6A–N). The results showed that in different subgroups, except for the subgroup with advanced N stage (N2-N3), there was no significant difference in overall survival between the high and low ERSS groups (Figure 6J, *p* = 0.137). In the other subgroups, compared with the low ERSS group, the high ERSS group had a lower overall survival rate (*p* < 0.05).

### 3.9 Analysis of tumor immune microenvironment

**FIGURE 7:**
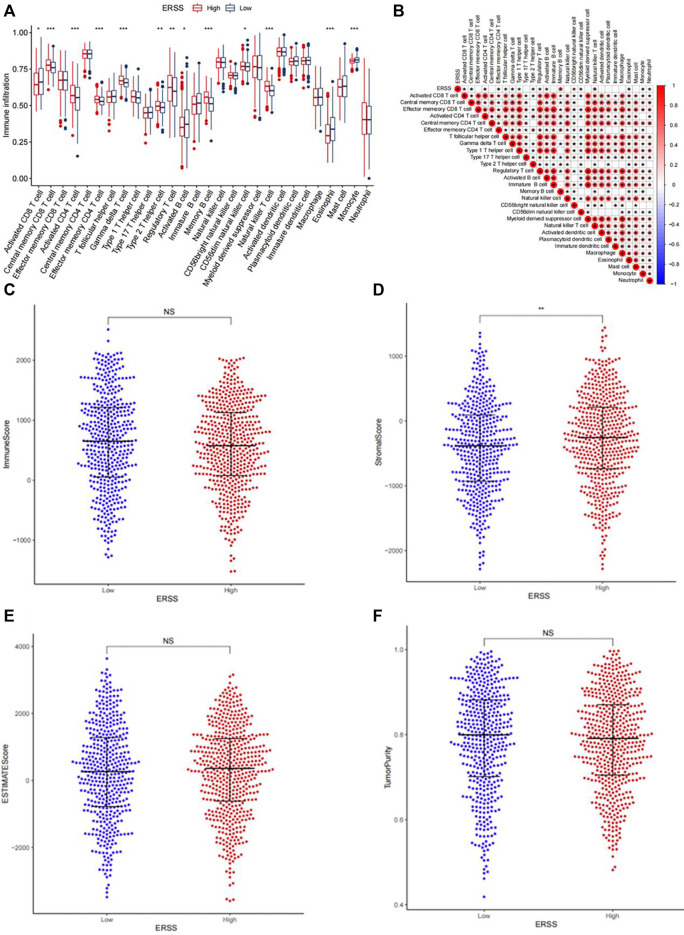
Analysis of tumor immune infiltration. **(A)** Infiltration of 28 types of immune cells in patients with high and low ERSS. **(B)** Heatmap of the correlation between ERSS and immune cell infiltration. **(C)** Comparison of immune scores between high and low ERSS patients. **(D)** Comparison of stroma scores between high and low ERSS patients. **(E)** Comparison of estimate scores between high and low ERSS patients. **(F)** Comparison of tumor purity between high and low ERSS patients; Where “*” represented *p* < 0.05, “**” represented *p* < 0.01, and “***” represented *p* < 0.001.

In order to explore the correlation between ERSS and the tumor immune microenvironment, this study evaluated the infiltration of 28 immune cells in each NSCLC patient based on the ssGSEA. The results showed (Figure 7A) that in the group with higher ERSS, central memory CD8 T cell, activated CD4 T cell, effector memory CD4 T cell, gamma delta T cell, type 2 helper T cell, regulatory T cell, memory B cells, CD56dim natural killer cell, and natural killer T cell were more abundant in infiltration; The group with lower ERSS showed more infiltration of activated CD8 T cell, activated B cell, eosinophil, and monocyte. As shown in Figure 7B, the correlation between ERSS and memory B cells was highest (R = 0.44, *p* < 0.05), followed by natural killer T cells (R = 0.34, *p* < 0.05). Meanwhile, compared to the low ERSS group, patients in the high ERSS group showed more abundant adaptive immune cell infiltration (Figure 7A), but did not show a matching survival advantage (Figure 1C), possibly because these immune cells were retained in the stroma surrounding tumor cells rather than penetrating their parenchyma (Chen and Mellman, 2017), Consistent with this, the results of ESTIMATE (Figure 7C–F) demonstrated that the group with high ERSS had higher stroma scores, and the difference was significant (Figure 7D, *p* < 0.01). These results suggested that activation of stroma in the tumor immune microenvironment may to some extent inhibit the function of adaptive immune cells.

### 3.10 Tumor mutation burden analysis

**FIGURE 8:**
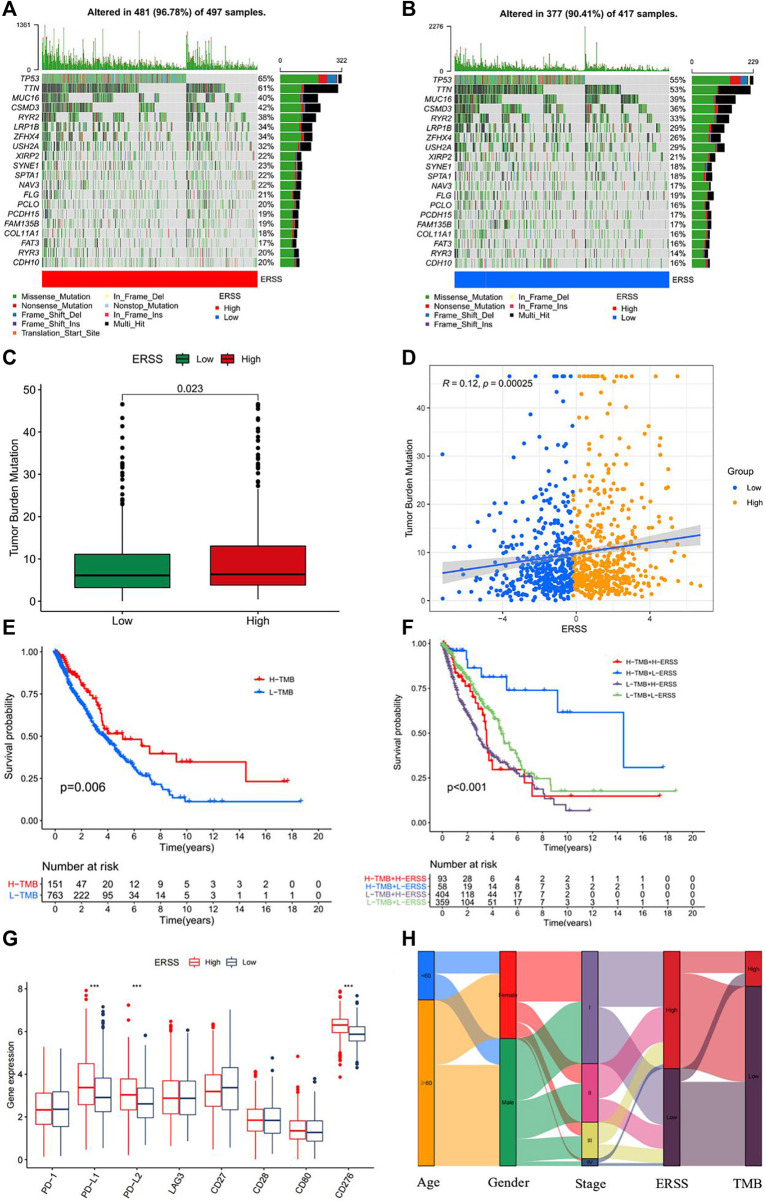
Tumor mutation burden analysis. **(A)** Somatic mutation frequency in patients with high ERSS. **(B)** Somatic mutation frequency in patients with low ERSS. **(C)** Box plot of TMB comparison between high and low ERSS patients. **(D)** Correlation plot between ERSS and TMB. **(E)** KM curve of patients with different TMB. **(F)** KM curve of composite TMB and ERSS. **(G)** The expression level of immune checkpoint genes in high and low ERSS patients. **(H)** Patient features Sankey diagram.

Research had shown that TMB was a key indicator for predicting clinical outcomes improvement in immunotherapy for NSCLC patients (Negrao et al., 2021; Ricciuti et al., 2022). In order to discuss the practical possibility of using ERSS in immunotherapy for NSCLC patients, the TMB of NSCLC patients in different ERSS groups was calculated. The results showed (Figures 8A, B) that the frequency of somatic gene mutations in the high and low ERSS groups was 96.78% and 90.41% respectively. After Wilcoxon’s test, the difference in TMB between the two groups was statistically significant (Figure 8C, *p* < 0.05), and there was a weak positive correlation between ERSS and TMB (Figure 8D, R = 0.12, *p* < 0.001). Meanwhile, this study also found that NSCLC patients with high TMB in the dataset achieved better survival outcomes (Figure 8E). In addition, regardless of TMB grouping, the overall survival rate of patients in the low ERSS group was significantly higher than that in the high ERSS group, and NSCLC patients with both low and high TMB characteristics had the highest overall survival (Figure 8F). Comparing the expression of commonly used immune checkpoint genes, the results showed that the expression of PD-L1, PD-L2, and CD276 was significantly increased in the high ERSS group (Figure 8G, *p* < 0.001). The above results clearly demonstrated that NSCLC patients with high ERSS were more likely to benefit from immunotherapy. The changes and associations in patients attribute features can be displayed using a Sankey diagram (Figure 8H).

### 3.11 Establishment and verification of nomogram

**FIGURE 9:**
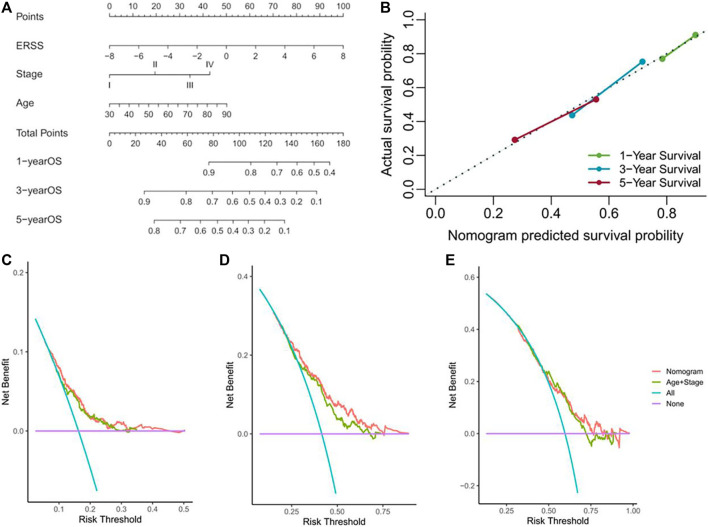
Establishment and verification of nomogram. **(A)** Nomogram for predicting the overall survival of NSCLC patients. **(B)** Calibration gram of column chart. **(C)** The decision curve analysis of NSCLC patients at 1 year. **(D)** The decision curve analysis of NSCLC patients at 3 years. **(E)** The decision curve analysis of NSCLC patients at 5 years.

In order to further verify the prognostic value of ERSS after therapeutic lung surgery and predict the actual survival possibility of NSCLC patients in clinical practice, this study constructed a nomogram (Figure 9A) using ERSS combined with commonly used prognostic factors in clinical practice, including age and pathological stage. At the same time, a calibration Gram was drawn based on Bootstrap method and the C-index was calculated to verify the accuracy of the nomogram, As shown in Figure 9B, there was excellent consistency between the predicted and observed results of the nomogram and the overall survival at 1, 3, and 5 years after surgery. Among them, the C-index predicted by the nomogram was 0.6509 (95%CI = 0.6316-0.6702), which was significantly better than the C-index combined with age and pathological stage (0.62601, 95%CI = 0.6056-0.6466), indicating that the predictive value of combining ERSS on the overall survival of NSCLC patients was better than that of combining age and pathological staging alone. In addition, the decision curve analysis was used to compare the clinical net benefits brought to patients under different strategies. The results found that choosing the nomogram as a predictor of overall survival for 3 years resulted in significantly higher clinical net benefits than commonly used clinical strategies (Figure 9D), but there was no significant difference in clinical net benefits between 1 and 5 years (Figures 9C, E).

### 3.12 GSEA analysis

Due to the fact that the occurrence and development of NSCLC were the result of multiple factors conducting together, the impact of high ERSS on tumor features may involve multiple signaling pathways. This study used GSEA_ 4.1.0 software to explore the potential biological pathway of high ERSS in NSCLC patients. The results demonstrated that, in the high ERSS group, “starch and sucrose metabolism”, “pathways in cancer”, “positive regulation of ubiquitin protein transferase activity” and “mitotic sisters chromatid separation” were significantly enriched (NOM *p*-value <0.05, FDR q-value <0.25). These results suggested that the metabolism and division levels of cancer cells in the high ERSS group were significantly higher than those in the low ERSS group, which may be the reason why the high ERSS group has adverse clinical features.

### 3.13 ERSS and drug sensitivity analysis

Chemotherapy still played a crucial role in the treatment of NSCLC patients, but patients had slightly different sensitivities to multiple chemotherapy drugs. Therefore, based on gene expression data of NSCLC patients, this study investigated the correlation between the efficacy of common NSCLC drugs in patients with different ERSS groups. The results showed that the sensitivity of tinib drugs (Axitinib, Erlotinib,Gefitinib) commonly used to treat NSCLC did not differ between high and low ERSS groups (*p* < 0.05), but in cisplatin, gemcitabine, and paclitaxel, the IC50 of the high ERSS group was significantly reduced compared to the low ERSS group (*p* < 0.05), indicating that the high ERSS group was more likely to benefit from chemotherapy with the above three drugs. This indicated that the ERSS in this study may become a potential basis for selecting chemotherapy drugs in clinical practice.

### 3.14 Core gene screening and determination

**FIGURE 10:**
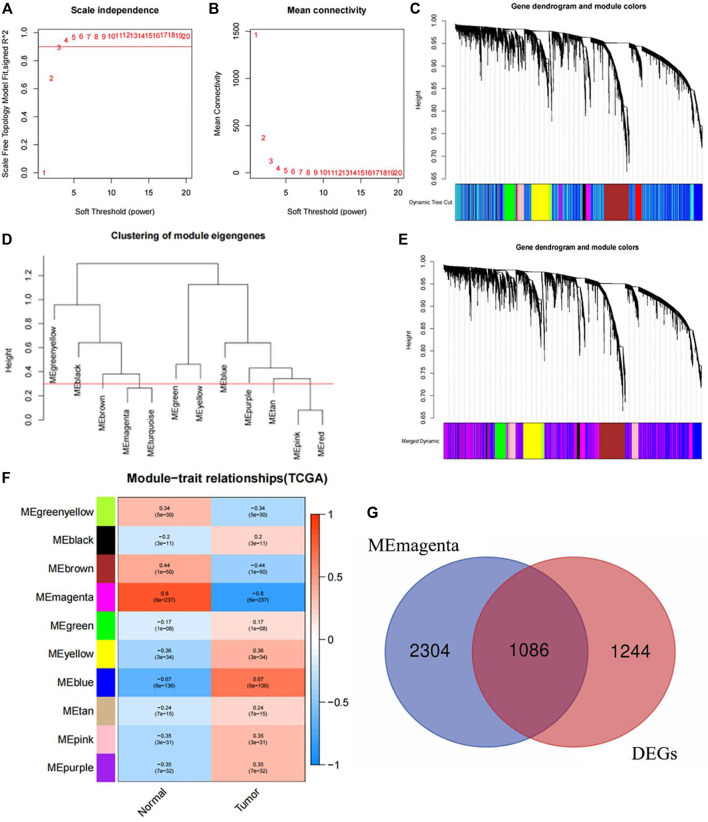
Core gene screening and determination with WGCNA. **(A)** Soft threshold under *R* ^2^. **(B)** The relationship between soft threshold and mean connectivity. **(C)** Dynamic tree. **(D)** The cutting height of each module. **(E)** The merged dynamic tree. **(F)** Correlation diagram between modules and traits. **(G)** Intersection diagram between Magenta module and different expressed genes.

Using WGCNA to construct a scale-free gene network for core gene screening, the results showed that when β = 3, *R* ^2^ reached 0.9, indicating good connectivity in the network (Figures 10A, B). Next, based on the calculated topological overlap matrix dissimilarity, gene hierarchical clustering was performed. 50 was used as the minimum number of genes in the module to obtain the dynamic tree (Figure 10C). Then, a pruning height of 0.3 was set to merge similar modules to obtain a merged dynamic tree consisting of 10 basic modules (Figure 10D, E). In order to study whether there was endoplasmic reticulum stress related gene in the core genes related to tumor occurrence, this study carried out correlation analysis on 10 gene modules and the feature about whether tumors or not. The results found that (Figure 10F) the Magenta module had the greatest correlation with tumor occurrence (|R| = 0.8, *p* < 0.001). 1086 differential core genes were obtained after intersection of Magenta module and different expressed genes (Figure 10G). After importing 1086 genes into STRING website to obtain their interaction network, and using the cytoHubba in Cytoscape_3.6.1 software to identify the core genes closely related to the occurrence of NSCLC. The results indicated that the endoplasmic reticulum stress-related gene CAV1, which was involved in constructing ERS, was in the top 15 positions among the 7 algorithms (BottleNeck, Closeness,Degree,EPC,MNC,Radiality, Stress), indicating that this endoplasmic reticulum stress-related gene called CAV1 may be closely related to the occurrence of NSCLC.

### 3.15 Clinical features of the research objects

The clinical features of the research subjects participating in the survey analysis were shown in Supplementary Table S3. A total of 862 eligible research subjects were included in this study, including 467 NSCLC cases and 395 controls. As stated in the inclusion and exclusion criteria, all research subjects, whether NSCLC patients or controls, were non-smokers in the Northeast Han female population. The data in the table indicated that the pathological type of NSCLC in non-smoking female was still mainly LUAD, with a total of 371 cases accounting for 79.4% of all cases, while LUSC was still relatively rare, with only 96 cases accounting for 20.6%. The average age of NSCLC patients was 56.8 ± 11.4, while the average age of the control group was 56.1 ± 11.6. After normality test, it was found that the age distribution of patients did not meet normality (*p* < 0.05). After comparing using Wilcoxon test, it was found that there was no significant difference in age distribution between the two groups (*p* = 0.398); After applying the Chi-square test analysis to other classification features, except for the uneven distribution of cooking oil exposure between the two groups (*p* < 0.05), the difference in the composition ratio of other features between the two groups was not statistically significant (family history of cancer: *p* = 0.221, passive smoking: *p* = 0.540). This indicated that the research subjects included in the study were from the same population, and the balance between the case group and the control group was well.

### 3.16 Polymorphism distribution and Hardy Weinberg equilibrium test of CAV1 rs3779514

The distribution of the polymorphism of CAV1 rs3779514 in the NSCLC case group was as follows: 433 (92.7%) with CC and 34 (7.3%) with CT + TT; In the control group, there were 349 (88.4%) with CC and 46 (11.6%) with CT + TT. In addition, a Hardy Weinberg test was performed on rs3779514, and the calculated distribution of each genotype showed that it conformed to the law of genetic balance (χ^2^ = 1.415, *p* = 0.493), which can be used for the next stage of analysis.

### 3.17 Association between CAV1 rs3779514 polymorphism and susceptibility to NSCLC

For the rs3779514 locus on the CAV1, this study used the CC as the wild reference group and CT + TT as the mutant genotype, and calculated the OR value and 95% CI of this locus using unconditional logistic regression to indicate the susceptibility to NSCLC. At the same time, to exclude the influence of age on NSCLC susceptibility, the age adjusted OR and 95% CI were also output simultaneously. As shown in Supplementary Table S4, compared with the study subjects carrying the CC wild-type genotype at the rs3779514 locus, non-smoking females carrying the mutated genotype CT + TT at this locus had a 40.4% reduced risk of developing NSCLC (OR = 0.596, 95% CI = 0.374-0.949, *p* = 0.029), and this significant trend in reducing susceptibility to NSCLC persisted after age adjustment (adjusted OR = 0.597, 95% CI = 0.375-0.951, *p* = 0.030). Based on the results of the allele model, it was also shown that compared to individuals carrying the C locus in the study subjects, individuals carrying the rs3779514T locus were only 59.7% more susceptible to NSCLC than the former (OR = 0.597, 95% CI = 0.380-0.938, *p* = 0.025). This indicated that the polymorphism at the rs3779514 locus in the CAV1 gene played a crucial role in the susceptibility to NSCLC in non-smoking females, and the rs3779514 mutation is a protective factor for non-smoking individuals who are female.

### 3.18 Clinical subgroup analysis of CAV1 rs3779514 polymorphism

#### 3.18.1 Analysis of CAV1 rs3779514 polymorphism and susceptibility to different pathological types of NSCLC

Due to the possible differences in genetic changes among different pathological types of NSCLC, a separate analysis was conducted on the association between CAV1 rs3779514 polymorphism and susceptibility to LUAD and LUSC by stratifying different pathological types in NSCLC. As shown in Supplementary Table S4, in LUAD, the susceptibility to LUAD was only 0.692 times in the study subjects carrying the CC + CT mutant genotype at the rs3779514 compared to the reference genotype. Unfortunately, this protective effect on the risk of developing LUAD did not show statistical significance (OR = 0.692, 95% CI = 0.428-1.117, *p* = 0.132), even after age adjustment, this regrettable situation had not changed (adjusted OR = 0.692, 95% CI = 0.429-1.118, *p* = 0.133), and there was still a statistically insignificant protective effect in the rs3779514 allele model (T/C) of LUAD (OR = 0.689, 95% CI = 0.433-1.097, *p* = 0.117). However, in LUSC, compared with the study subjects carrying the CC genotype at the rs3779514, the subjects carrying the mutated genotype CT + TT at this locus had a cancer risk of only 0.245 times that of the former (OR = 0.245, 95% CI = 0.074-0.805, *p* = 0.020). After adjusting by the age, they still had a strong effect on reducing the risk of LUSC occurrence (adjusted OR = 0.249, 95% CI = 0.076-0.821, *p* = 0.022). In addition, the allele model (T/C) results at the rs3779514 in LUSC showed that the T locus had a strong effect on reducing the susceptibility to LUSC in the study subjects (OR = 0.251, 95% CI = 0.077-0.815, *p* = 0.021).

#### 3.18.2 Analysis of CAV1 rs3779514 polymorphism and susceptibility to NSCLC in different age groups

Due to the increased risk of lung cancer in the majority of the population after the age of 60, this study chose to stratify the study subjects based on different age states, using the age limit of 60. The association between the CAV1 rs3779514 polymorphism and the risk of NSCLC in the age group <60 years old and the ≥60 years old were analyzed separately. As shown in Supplementary Table S4, in the age group <60 years old, for the rs3779514 in CAV1, the study subjects carrying the CT + TT genotype showed a lower likelihood of developing NSCLC compared to the CC genotype, with a 54.7% lower risk of developing NSCLC (OR = 0.453, 95% CI = 0.238-0.863, *p* = 0.016). In addition, the risk of NSCLC in individuals carrying the rs3779514T was 0.454 times compared to that carrying the rs3779514 C (OR = 0.454, 95% CI = 0.243-0.847, *p* = 0.013). However, in the age group ≥60 years old, although the mutated genotype (CT + TT/CC) of rs3779514 reduced the susceptibility to NSCLC to some extent, the reduction in risk was not statistically significant (OR = 0.821, 95% CI = 0.414-1.631, *p* = 0.574), and even in the analysis of the rs3779514 allele model (T/C), the non-significance of this risk correlation could not be reversed (OR = 0.829, 95% CI = 0.425-1.619, *p* = 0.583).

#### 3.18.3 Analysis of CAV1 rs3779514 polymorphism and susceptibility to NSCLC with a family history of cancer

Family clustering was a prominent characteristic in the occurrence of cancer. In order to avoid family history of cancer becoming a confounding factor, this study stratified different cancer family histories in NSCLC and analyzed the association between CAV1 rs3779514 polymorphism and the risk of NSCLC occurrence in the study subjects with or without a family history of cancer. As shown in Supplementary Table S4, in the population with a family history of cancer, the susceptibility to NSCLC in the study subjects carrying the mutated genotype (CT + TT) at rs3779514 increased compared to those with genotype CC, regardless of age adjustment. However, this increased risk was not statistically significant (OR = 2.636, 95% CI = 0.260-26.760, *p* = 0.412; adjusted OR = 1.774, 95% CI = 0.164-19.221, *p* = 0.637), The same conclusion can also be obtained in the allele model analysis of the rs3779514 (T/C) (OR = 2.565, 95% CI = 0.260-25.324, *p* = 0.420). The reason for the opposite trend between the above and the rest of the conclusions may be due to the relatively small number of subjects with a family history of cancer in the study, resulting in a certain degree of volatility in this stratification. The conclusions were not statistically significant and had little reference significance. However, in a population without a family history of cancer, the mutated genotype (CT + TT) at the rs3779514 reduced the susceptibility to NSCLC to some extent compared to the study subjects carrying the CC genotype. Unfortunately, the results were not significant (OR = 0.729, 95% CI = 0.396-1.344, *p* = 0.311; adjusted OR = 0.727, 95% CI = 0.394-1.342, *p* = 0.308), and the study subjects carrying the rs3779514 C allele as a reference also found a statistically insignificant trend in reducing the risk of NSCLC occurrence in the study subjects carrying the T allele (OR = 0.742, 95% CI = 0.410-1.345, *p* = 0.326).

#### 3.18.4 Analysis of CAV1 rs3779514 polymorphism and susceptibility to NSCLC with different passive smoking conditions

Passive smoking also played a crucial role in the occurrence of lung cancer. In order to reduce the confounding bias caused by passive smoking in the analysis of this study, the association between the CAV1 rs3779514 polymorphism and the susceptibility to NSCLC in study subjects with different passive smoking conditions was separately analyzed after stratification. As shown in Supplementary Table S4, in the passive smoking group, the study subjects carrying the rs3779514 mutant (CT + TT) showed a lower susceptibility to NSCLC compared to those with the CC genotype. Despite age adjustment, they still had the same conclusion, but unfortunately did not have the significance (OR = 0.717, 95% CI = 0.329-0.1563, *p* = 0.403; adjusted OR = 0.701, 95% CI = 0.321-1.533, *p* = 0.374), the allele model (T/C) also clearly showed no significant results (OR = 0.730, 95% CI = 0.342-1.558, *p* = 0.416). Similarly, in the group without passive smoking, no significant conclusions were drawn regarding the association between CAV1 rs3779514 polymorphism and susceptibility to NSCLC (CT + TT/CC: OR = 0.892, 95% CI = 0.374-2.128, *p* = 0.797; adjusted OR = 0.885, 95% CI = 0.369-2.118, *p* = 0.783; T/C: OR = 0.897, 95% CI = 0.385-2.091, *p* = 0.802).

### 3.19 Analysis of the susceptibility of NSCLC to the interaction between CAV1 rs3779514 polymorphism and cooking oil exposure

Considering that the clinical characteristic analysis results of the study subjects showed that the proportion of cooking oil exposure in the cases and the controls was different (Supplementary Table S3, *p* < 0.05), and the relationship between the susceptibility to NSCLC and cooking oil exposure was also obtained through logistic regression (adjusted β (Se) = 0.590 (0.196), OR = 1.803, 95% CI = 1.229-2.645, *p* = 0.003), thus summarizing the conclusion that the increased risk of NSCLC was closely related to exposure to cooking fumes. Based on a comprehensive consideration of the significance of the interaction between genetic susceptibility and environmental factors in disease occurrence, this study conducted an analysis of the interaction between CAV1 rs3779514 polymorphism and exposure to cooking oil. This study obtained a total of 507 study subjects who had the information about cooking oil exposure, including 154 exposed individuals and 353 non exposed individuals (Supplementary Table S3). As the T site of CAV1 rs3779514 was a protective factor, we used mutated individuals carrying the T site (CT + TT) and those who did not have exposure to cooking oil as the reference population, and applied crossover analysis. As shown in Supplementary Table S5, the susceptibility to NSCLC with CC genotype at the CAV1 rs3779514 and simultaneous exposure to cooking oil was 2.233 times higher than that without cooking oil exposure and carrying CT + TT genotype at the CAV1 rs3779514 (OR = 2.233, 95% CI = 1.054-4.732, *p* = 0.036), and even increased after age adjustment (adjusted OR = 2.254, 95% CI = 1.063-4.781, *p* = 0.034). This suggested that there was a possible interaction between the CAV1 rs3779514 and the cooking oil exposure.

Next, based on the results of crossover analysis and the automated calculation table developed by Tomas Andersson, the additive effect of CAV1 rs3779514 and cooking oil exposure could be analyzed. In the additive model, the 95% CI of RERI was (−1.304, 2.235), and the 95% CI of AP was (−0.591, 1.004), with 0 included in the 95% CI; The 95% CI of S was (0.149, 17.002), which included 1. All three results indicated that there was no additive interaction between the two. Meanwhile, the multiplication model analyzed the interaction between CAV1 rs3779514 and cooking oil exposure, and the results found that the interaction term in the multiplication model was still not statistically significant (OR = 1.173, 95% CI = 0.335-4.112, *p* = 0.803; adjusted OR = 1.176, 95% CI = 0.335-4.126, *p* = 0.800), indicating that there was also no multiplication interaction between CAV1 rs3779514 and cooking oil exposure.

### 3.20 Analysis of the prognostic role of CAV1 rs3779514 polymorphism in NSCLC patients

#### 3.20.1 Kaplan-Meier survival analysis

After following up on NSCLC patients included in this study and excluding data with incomplete information and logical errors, a total of 212 follow-up data were obtained. Draw Kaplan Meier curves based on the CAV1 rs3779514 genotype, survival time, and survival status of the follow-up study subjects, and compare the differences in overall survival among different genotypes. There was no statistically significant difference in overall survival between CC and CT + TT in CAV1 rs3779514 (*p* = 0.662). According to the early pathological stage (I-II) and late pathological stage (III-IV), Kaplan Meier curves were plotted for each subgroup, no statistical difference in overall survival between different genotypes of CAV1 rs3779514 was observed in both subgroups (*p* = 0.579, *p* = 0.461).

#### 3.20.2 Cox regression analysis of CAV1 rs3779514 polymorphism and clinical features

Next, Cox regression analysis used to identify factors that affect the prognosis of non-smoking female NSCLC patients. In order to avoid collinearity between variables and ensure that as many variables as possible were included in the analysis, TNM stages included in the model. In univariate Cox regression analysis, the overall survival of non-smoking female with NSCLC was significantly correlated with age, T stage, N stage, M stage and pathological stage (*p* < 0.05), but no prognostic effect of CAV1 rs3779514 polymorphism was observed (*p* = 0.652). The statistically significant variables in univariate Cox regression analysis were included in multivariate Cox regression analysis. The results were shown, T stage, N stage, M stage, and pathological stage were all independent prognostic factors, and the risk of death in non-smoking female NSCLC patients significantly increased with stage progression (HR > 1, *p* < 0.05).

## 4 Discussion

For NSCLC patients, early diagnosis was crucial because the overall survival of advanced stage patients was poor, with over half of patients dying within 3 months of diagnosis (Snee et al., 2021). Even though systemic anti-cancer treatment can save the patient’s survival outcome to some extent, the proportion of patients participating in systemic anti-cancer treatment was still very low, so the prognosis of advanced stage NSCLC patients was still poor (Snee et al., 2021). Due to the high heterogeneity of tumors, developing more effective clinical treatment strategies required a deep understanding of the factors that affect the occurrence and development of NSCLC. New biomarkers would be expected to become new therapeutic targets with the rapid development of bioinformatics and cell sequencing. In recent years, various biological signaling pathways induced by protein homeostasis imbalance caused by endoplasmic reticulum stress had become important markers for various cancers, including lung cancer (Yao et al., 2020; Yoon et al., 2021; Yu et al., 2023a). The role of endoplasmic reticulum stress in the occurrence and development of NSCLC is complex. On the one hand, studies have shown that endoplasmic reticulum chaperone binding proteins can mediate extracellular PKM2 to promote the migration and invasion of NSCLC (Zhang et al., 2022), On the other hand, activation of the endoplasmic reticulum stress signaling pathway can significantly inhibit NSCLC cell viability and promote cell apoptosis (Hsu et al., 2017), This may depend on the pathway, intensity, and duration of endoplasmic reticulum stress activation. Although the relationship between endoplasmic reticulum stress and NSCLC had been extensively explored in cell experiments, each experiment usually only involved fewer endoplasmic reticulum stress-related genes. Therefore, the biological significance of more endoplasmic reticulum stress-related genes on the overall characteristics of NSCLC deserved further research.

This study constructed a prognostic feature ERSS based on 14 endoplasmic reticulum stress-related genes selected, and divided ERSS into two groups according to the optimal cutoff value. The relationship between ERSS and clinical features and overall survival was validated in external validation dataset. After testing, it was found that ERSS was more expressed in male, smoking, and more invasive clinical features (Figure 2; Figure 5), and was an independent prognostic factor, significantly increasing the risk of patient death (Figures 3B, D; Figure 6P). As an independent prognostic factor of NSCLC, it may involve a variety of reasons. In terms of biological mechanisms, the endoplasmic reticulum is the main site of protein synthesis, folding and modification in the cell. In malignant tumor cells, due to the influence of many factors, the function of the endoplasmic reticulum may be disturbed, which leads to endoplasmic reticulum stress. Endoplasmic reticulum stress can trigger a series of signaling pathways, which may ultimately affect cell survival, metastasis, etc. Most cancer-related mutations in IRE1α disable their apoptotic output, which may lead to the shutdown of terminal UPR, allowing cancer cells to survive (Xue et al., 2011; Ghosh et al., 2014). And IRE1α can regulate cytoskeleton remodeling and cell migration by directly binding to filamin A to control cytoskeleton dynamics and cell motility (Urra et al., 2018). In addition, autophagy induction in tumors exposed to hypoxia and/or low nutrient conditions may provide an energy source that promotes cancer cell survival and resistance to chemotherapy (Jiang et al., 2015). From another perspective, several key genes that construct ERSS also play an important role in the occurrence and development of lung cancer. SLC2A1 expression and glucose metabolism were increased in tumor-associated neutrophils from mouse models of lung adenocarcinoma compared with normal neutrophils, and tumor growth was slowed and radiotherapy effects were enhanced when SLC2A1 expression was absent in tumor-associated neutrophils (Ancey et al., 2021). TGM2 plays a role in promoting TRAIL resistance and cell migration through upregulation of c-FLIP and MMP-9, respectively (Li et al., 2011). Interdependent positive regulation of GJB2 and PI3K/Akt pathways promotes gefitinib resistance in NSCLC by inducing EMT (Yang et al., 2015). SAM68 drives cancer metabolism by mediating alternative splicing of pyruvate kinase (PKM) pre-mRNAs and promoting the formation of PKM2 (Zhu et al., 2021). ERO1A can reshape TME to drive sustained immunosuppression maintained by endoplasmic reticulum stress response, thereby influencing the immune environment and response to PD-1 blockade, contributing to tumor survival (Liu et al., 2023). Increased GAPDH expression is associated with the proliferation and invasion of lung cancer (Hao et al., 2015). High PTGIS is associated with poorer overall survival and progression-free survival of lung cancer (Dai et al., 2020). CP is produced heterotopically in lung adenocarcinoma cells, and its expression correlates with tumor progression (Matsuoka et al., 2018). CKAP4 promotes cancer cell proliferation through the PI3K/AKT pathway (Kimura et al., 2016). Interestingly, THBS1 inhibits exosom-induced lung cancer cell migration and invasion (Huang et al., 2019a). Downregulation of NUPR1 gene expression can limit the growth of human non-small cell lung cancer *in vivo* and *in vitro* (Guo et al., 2012). Overexpression of CAV1 can significantly inhibit the proliferation rate of lung adenocarcinoma cells (Yan et al., 2019). In recent years, the complex tumor microenvironment composed of various non tumor cells surrounding tumor cells had received widespread attention from researchers, as promoting proliferation, invasion and metastasis, resisting apoptosis, inducing abnormal blood supply angiogenesis, and immune escape all depended to a certain extent on the tumor microenvironment, playing a key role in cancer progression and drug resistance (Xiao and Yu, 2021). Therefore, this study evaluated immune cell infiltration and overall stroma score in different ERSS groups using ssGSEA and ESTIMATE. The results showed that there was a higher level of immune cell infiltration in the high ERSS group (Figure 7A), but it did not seem to have anti-tumor effects and still had a higher probability of death (Figure 1C). Consistent with this result, experimental studies have found that the surrounding stroma of lung tumor cells was not only a more favorable environment for attracting T cells, but also a dense network of stroma fibers that restricted T cells from entering tumor cells (Salmon et al., 2012). At the same time, there was also a specific adaptive immunosuppression phenomenon that worked at the tumor microenvironment level in metastatic melanoma (Gajewski et al., 2017), this study to some extent supported the above research findings. In addition, immunotherapy improves the therapeutic effect of advanced NSCLC by activating the host immune system and regulating the tumor microenvironment (Leonetti et al., 2019), moreover, meta-analysis showed that NSCLC patients with high TMB significantly improved overall survival after treatment with immune checkpoint inhibitors (Nan et al., 2021). Therefore, this study analyzed TMB and immune checkpoints, and the results showed a significant positive correlation between the expression of ERSS and TMB and commonly used immune checkpoints such as PD-L1 and PD-L2 (Figure 8), indicating that ERSS may also become a biomarker for predicting immunotherapy prognosis and clinical benefits.

Meanwhile, research had shown that, compared with conventional clinical strategies, combining the characteristics of ERSS had become a more suitable model for predicting the overall survival of NSCLC patients. The decision curve drawn can compare the clinical net benefits of different plans based on different risk thresholds, in order to choose a more effective prediction plan in actual clinical work. Next, in order to explore the potential functional mechanism of the high ERSS group, this study conducted a GSEA analysis, and the results showed that, compared with the low ERSS group, the high ERSS group showed significantly activated “starch and sucrose metabolism”, “positive regulation of ubiquitin protein transferase activity” and “mitotic sisters chromatid separation” signal pathways. The rapid proliferation of tumors was closely related to the adaptive changes in cellular metabolic pathways, and many studies had found that active sugar metabolism in tumors played a driving role in the malignant characteristics of tumors (Tang et al., 2021), meanwhile, sugar metabolism in cancer cells provides metabolic flux for DNA synthesis and damage repair, thereby promoting cancer cell survival and metastasis as well as resistance to radiation therapy (Yu et al., 2023b). The upregulated ubiquitin proteasome system in cancer cells can lead to targeted degradation of various tumor suppressor factors such as p53, which is beneficial for the survival of cancer cells (Aliabadi et al., 2021). In addition, mitotic sisters chromatid separation occurred at the late stage of mitosis, and this process was significantly enriched, suggesting faster mitosis. The incorrect separation mechanism during cell division can cause genomic instability, becoming one of the factors leading to the occurrence and development of cancer (Jamasbi et al., 2022). Researches on these signaling pathways may to some extent explain why the high ERSS group had adverse clinical and prognostic characteristics compared to the low ERSS group. Then, based on the patient’s gene expression data, the sensitivity of each patient to different drugs was predicted and compared in different ERSS groups, which may help in individualized clinical drug selection.

The single nucleotide polymorphism of genetic material was still closely monitored by researchers from various countries, and it had been found to be closely related to the susceptibility of NSCLC (Dai et al., 2019; Pineda Lancheros et al., 2022). After preliminary analysis, this study found that CAV1 may be a core gene in the occurrence of NSCLC. Based on a clear exploration of the relationship between genes and NSCLC while excluding the interference of smoking as an environmental factor, this study selected non-smoking females who may be more susceptible to genetic susceptibility as the research subjects to analyze the core role of CAV1 single nucleotide polymorphism in the occurrence and development of NSCLC.

At present, multiple studies had focused on analyzing the role of CAV1 in NSCLC, but interestingly, the function of CAV1 in NSCLC was still controversial, and the results obtained by various studies were not the same. Wei’s research showed that the expression of CAV1 was significantly reduced in NSCLC, and overexpression of CAV1 can reduce cell migration by affecting the phosphorylation of STAT3, exerting a tumor suppressive effect (Peng et al., 2019). However, Huang’s experiments found that upregulation of CAV1 expression can trigger a certain degree of cisplatin resistance in NSCLC (Huang et al., 2019b), Dominic’s research also found that knocking out CAV1 can restore radiosensitivity in NSCLC cells exhibiting radiation resistance (Leiser et al., 2021), it was clearly stated that CAV1 had a certain degree of protective effect on NSCLC cells. At the same time, not only was the double-sided features of CAV1 discovered in cytology experiments, but a meta-analysis involving 1513 study subjects also reached the same conclusion. The analysis showed that CAV1 plays an inhibitory and promoting role in the occurrence and progression of NSCLC, respectively. This effect was specifically reflected in: firstly, the expression of CAV1 in human NSCLC was significantly lower than in normal tissues, indicating that the absence of CAV1 can lead to tumorigenic transformation of normal cells; Secondly, the positive expression of CAV1 leaded to adverse prognosis in NSCLC patients, indicating that CAV1 played a positive promoting role in tumor progression (Chen et al., 2014). However, most of the analysis on CAV1 and NSCLC focused on the effects caused by changes in CAV1 expression, and the analysis of single nucleotide polymorphism of CVA1 in NSCLC was still insufficient. Therefore, it was still necessary to analyze the role of single nucleotide polymorphism of CAV1 in NSCLC.

In order to explore the relationship between susceptibility to NSCLC in non-smoking women with CAV1 single nucleotide polymorphism, this study combined the Ensemble database’s CAV1 polymorphism data from the reference population in China and Haploview 4.2 software to select tag locus. After inclusion, exclusion, and preliminary analysis, the rs3779514 that met the requirements was selected. rs3779514 was located in the intron region of CVA1, and according to the functional prediction database rSNPBase (Guo and Wang, 2018), it was suggested that rs3779514 was an expression quantitative trait locus (eQTL) in CAV1. Therefore, exploring this locus may have certain scientific value. This study conducted a comprehensive and stratified analysis for the susceptibility to NSCLC of non-smoking females at the CVA1 rs3779514 locus for the first time. The results showed that the rs3779514 mutation significantly reduced the overall susceptibility to NSCLC in the study subjects (Supplementary Table S4: adjusted OR = 0.597, 95% CI = 0.375-0.951, *p* = 0.030). In the stratified analysis, the rs3779514 mutation still showed some significant changes in the risk of NSCLC occurrence, the probability of developing NSCLC decreased in the study subjects with mutated genotypes in the LUSC group (Supplementary Table S4: adjusted OR = 0.249, 95% CI = 0.076-0.821, *p* = 0.022) and the age group below 60 (Supplementary Table S4: OR = 0.453, 95% CI = 0.238-0.863, *p* = 0.016). Unfortunately, there was no correlation between the CVA1 rs3779514 locus and the susceptibility to NSCLC in the stratification of family history of cancer and passive smoking (Supplementary Table S4). Due to the characteristic of eQTL interfering with gene expression and being associated with genetic polymorphism in complex diseases (Li et al., 2015). Therefore, the fundamental reason why non-smoking females carrying the rs3779514 mutation in the CAV1 gene had a lower susceptibility to NSCLC may be due to the role of eQTLs at this locus, which affected the expression of CAV1 and played a decisive role in whether normal cells can transform into cancer cells. In addition, due to the complex cooking patterns in our country, the likelihood of adult females being exposed to cooking oil for life increased, and exposure to cooking oil increased the risk of non-smoking females developing lung cancer by nearly twice the risk of non-exposed individuals (Jia et al., 2018). Therefore, this study analyzed the interaction between cooking oil exposure and rs3779514, and the results found that even though there may be an interaction between cooking oil exposure and rs3779514 in the crossover analysis (Supplementary Table S5), no significant results were obtained in the analysis of specific additive and multiplicative interaction models. This may be due to the presence of some lost individuals in the collection of cooking oil exposure, or more complex interactions which requires further investigation in epidemiological surveys that include more research subjects. Finally, based on the collected follow-up information, Kaplan Meier survival curves and Cox regression analysis were performed on the study subjects, but no impact of CVA1 rs3779514 on prognosis was observed. The genotype change at the CVA1 rs3779514 was only associated with the risk of NSCLC in non-smoking females and not with overall survival.

The ERSS established in this study based on endoplasmic reticulum stress-related genes uses a more novel principal component analysis construction method compared with other similar articles, and the ERSS constructed in this study has better predictive power compared with another similar study (Shen et al., 2022). In addition, we analyzed single nucleotide polymorphisms of CAV1, one of the key genes in ERSS construction, and found an association between changes in CVA1 rs3779514 genotype and the risk of NSCLC in non-smoking women. Moreover, the current analysis of CAV1 and NSCLC mostly focuses on the expression of CAV1, and there is a lack of analysis of NSCLC from other aspects of CAV1 (Kato et al., 2004; Liu et al., 2021). Therefore, the novelty of this study lies in the integration of genomic data and clinical data, the application of principal component analysis method, the construction of ERSS with certain predictive efficacy based on endoplasmic reticulum stress-related genes, and the single nucleotide polymorphism analysis of the key gene CAV1. It provides insights into the molecular mechanism of NSCLC, identifies potential prognostic markers, and enhances the understanding of the etiology of NSCLC, making the research more convincing and important in research value, and provides a novel research idea for reference, which is conducive to personalized medicine. However, there were still some shortcomings in this study. Firstly, the included research subjects were all from the Northeast region of China, which was subject to certain limitations when extrapolating conclusions; Secondly, as rs3779514 is a characteristic of eQTLs, the correlation between the genotype changes of rs3779514 and CAV1 expression was not observed. Further research and exploration are still needed in the future.

## Data Availability

The public data of this study came from TCGA (http://cancergemome.nih.gov/), Genecards (https://www.genecards.org/), and GEO (http://www.ncbi.nlm.nih.gov/geo/) databases. The rest of the data is not available due to patient privacy and confidentiality.

## Supplementary material

**SUPPLEMENTARY TABLE S1**
Distribution of ERSS in different clinical features of NSCLC patients.

**SUPPLEMENTARY TABLE S2**
Distribution of ERSS in different clinical features of NSCLC patients in the GEO validation dataset.

**SUPPLEMENTARY TABLE S3**
Clinical features of research subjects.

**SUPPLEMENTARY TABLE S4**
Relationship between rs3779514 polymorphism and risk of NSCLC occurrence.

**SUPPLEMENTARY TABLE S5**
Crossover analysis of rs3779514 polymorphism and exposure to cooking oil.
